# The interactive roles between coping tendency and focus on COVID-19 information time in Adolescent Obesity

**DOI:** 10.1186/s40359-025-03766-x

**Published:** 2025-12-11

**Authors:** Nannan Wu, Guoli Yan, Yan Zou, Ruochen Yan, Ling Sun, Min Hou, Huifang Yin, Guangming Xu

**Affiliations:** 1 Department of Laboratory Medicine, Tianjin Chest Hospital, Tianjin, China; 2 Mental Health Center of Tianjin Medical University, Tianjin Anding Hospital, Tianjin, China; 3 Mental health center, Civil Aviation University of China, Tianjin, China

**Keywords:** Adolescent obesity, Coping tendency, Interactive roles, COVID-19 information time

## Abstract

**Aim:**

This study examined the relationship between adolescents’ coping tendency, the focus on COVID-19 information time, and adolescent obesity during the COVID-19 pandemic.

**Methods:**

A prevalence survey of 13,374 students from 13 secondary schools in Tianjin was conducted between April and June 2022 using convenience cluster sampling. Basic information on the adolescents’ heights, weights, and psychological health conditions was collected using a general information questionnaire and a coping style questionnaire.

**Results:**

Obese adolescents scored significantly lower on positive coping styles. In addition, adolescents who spent more than three hours on COVID-19 information had a significantly higher rate of obesity than the other groups. The findings of the regression analysis indicate that a positive coping style (0.980, 0.974–0.986) functions as a protective factor, while the amount of time spent on COVID-19 information (1.140, 1.088–1.195) serves as a risk factor. The findings indicate that the interaction between positive coping tendency and less time focus on COVID-19 information significantly influences adolescent obesity (0.923, 0.904–0.942).

**Conclusions:**

Increased daily time spent with COVID-19 information was associated with a higher incidence of adolescent obesity. Positive coping styles have been observed to reduce the incidence of obesity in adolescents. Positive coping tendency and less time focus on COVID-19 information can mutually reinforce the reduction in adolescent obesity. In developing interventions for adolescent obesity during periods of chronic crisis, authorities must focus on enhancing adolescents’ coping mechanisms and reducing the amount of time spent focusing on crises.

**Supplementary Information:**

The online version contains supplementary material available at 10.1186/s40359-025-03766-x.

## Introduction

 Current socioeconomic development has changed the dietary patterns and lifestyles of adolescents. The increasing prevalence of overweightness and obesity among adolescents has become an independent risk factor for physical and mental health problems [1]. A biosocioecological model has been proposed as a potential explanatory framework for the prevalence of obesity among adolescents. The model under discussion integrates a range of factors, including public policies, society, community and built environment, childcare and school education, family and peer support, and personal factors [2].

On December 31, 2019, the World Health Organization classified the COVID-19 pandemic as a public health emergency of international concern [3]. During the COVID-19 pandemic, the potential risks of infection and extensive implementation of preventive and control measures, such as home quarantine, changed people’s lifestyles and dramatically impacted the mental and physical well-being of adolescents [4, 5]. Studies revealed a significant increase in obesity rates among adolescents during the COVID-19 pandemic [6, 7]. A study of 19,066 preschool children in China [8] showed an increase in childhood obesity rates from 10.47% to 12.28% before and after school closures. Another study of 10,082 youths in China [9] revealed that the overweight rate increased from 21.4% to 24.6% during school closures. The study concluded that lockdowns in schools and communities and social restrictions during the COVID-19 pandemic reduced opportunities for outdoor activities, leading to a sedentary lifestyle that caused weight gain [10]. Moreover, adolescents are susceptible to emotional eating when faced with high levels of stress, which further exacerbates weight gain [11]. Nakamura found that acute stress usually suppresses appetite [12], whereas chronic stress promotes the secretion of cortisol, leading to an increased drive to eat, which in turn promotes the need for and the behavior to seek out and consume high-fat and high-energy foods. During the pandemic, the public primarily accessed information from external media sources through electronic devices. Excessive information and screen time have been demonstrated to have detrimental effects on adolescents’ mental health and physical well-being, with studies showing an association between these factors, anxiety, and obesity [2, 13].

Individual psychological factors play a non-negligible role in the development of adolescent obesity, and coping styles are an important aspect of these factors. Coping styles refer to the cognitive and behavioral approaches that individuals adopt when faced with stressful situations or events [14]. They are categorized into positive and negative coping styles. The former includes seeking support and attempting to change, whereas the latter includes avoidance and venting [15]. Among studies focusing on the relationship between coping styles and obesity, Yang confirmed that positive coping styles were negatively associated with obesity [16]. Varela found that negative coping styles were positively associated with negative eating behaviors that led to obesity, such as emotional and restrained eating [17]. Zheng also confirmed that negative coping styles played a partial mediating role in the relationship between stressful life events and internalizing problems in overweight and obese adolescents and could exacerbate the development of adolescent obesity [18]. In the 2025 South China Expert Consensus on the Diagnosis and Management of Childhood and Adolescent Obesity, Tang explicitly stated that a positive attitude among adolescents with obesity could lead to weight loss [19]. In light of the research theories cited above, it is important to acknowledge the significance of positive coping strategies in adolescent obesity interventions.

Despite the findings of previous studies on the role of various factors in adolescent obesity during the COVID-19 pandemic, the specific effects of coping tendency and screen time on adolescent obesity remain ambiguous. The present study aimed to contribute to ongoing research on the effective reduction of the incidence of adolescent obesity and to identify key intervention factors. Therefore, the present study comprehensively considered various factors such as the amount of time spent on COVID-19 information, coping styles, anxiety, and eating patterns during the COVID-19 pandemic, and explored the mechanisms associated with adolescent obesity in chronic crisis events. The study investigated the potential association between coping tendency, the amount of time spent on COVID-19 information, and adolescent obesity. We hope that the findings of this study will be applied to future relevant crises and will provide a theoretical basis for intervention programs for adolescent obesity.

## Methods

The data for this study were obtained from the Tianjin Mental Health Promotion Program for Children and Adolescents

**Fig. 1:**
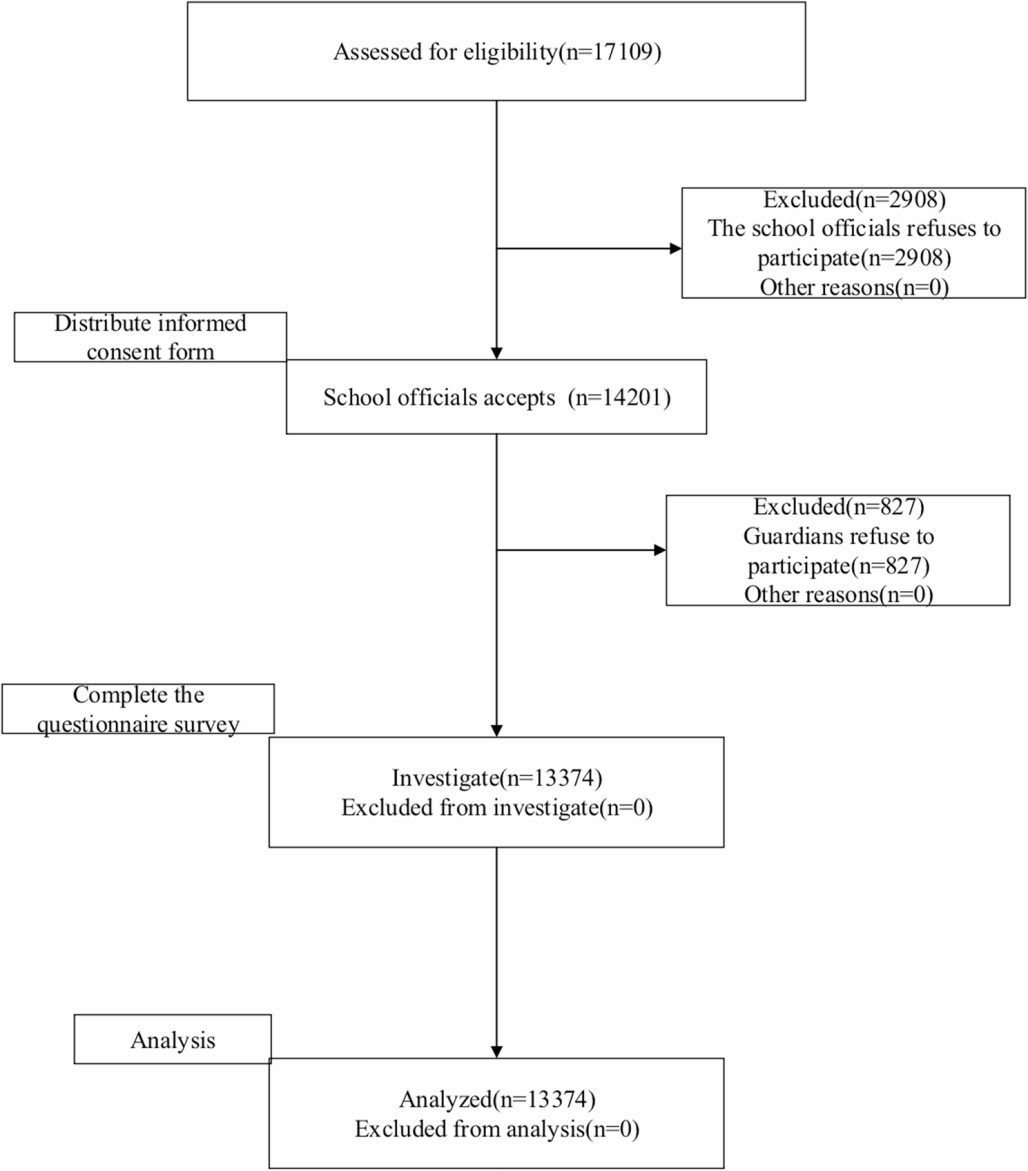
Sampling process diagram

We selected one district with 20 secondary schools and a total of 17,109 students from among the ten districts participating in the mental health promotion project. We informed the school officials about the investigation work, and 13 schools with a total of 14,201 students agreed to carry it out. After the guardians of 14,201 students provided informed consent, 13,374 people participated in the survey and were included in the analysis, giving an effective response rate of 94.2%(see Fig. 1).The inclusion criteria for the study were as follows: firstly, all current middle and high school students in the designated sampling area were to be included; secondly, the adolescents were to study remotely at home using online devices. The exclusion criteria were as follows: firstly, if guardians and teenagers refused to participate in the investigation; and secondly, if subjects suffered from severe mental or physical illnesses. Of the 13,374 students, 6,745 (50.4%) were male and 6,629 (49.6%) were female. Participants’ mean age was 15.21 ± 1.433 years, and the age range was 11–19 years. This study was conducted from April to June 2022, during which the students mainly studied online from home. All participating adolescents and their guardians provided written informed consent, and the adolescents and their guardians consented to the publishing of all images, clinical data, and other data included in the manuscript. This study was approved by the Medical Ethics Committee of Tianjin Anding Hospital (2021-42).

### Survey process

As a first step, the research team organized online training for the teachers responsible for the survey across participating schools. The purpose of the survey, precautions, ethical norms, and operational steps were explained, and the link to the online survey and informed consent form were sent to the teachers. The online survey was distributed through the QuestionStar assessment system. This system was developed by Changsha Ranxing IT, Ltd. The company is based in Changsha, Hunan Province, China. The project team signed a nondisclosure agreement with the company to ensure participants’ privacy and security. In the second step, the teachers responsible for the survey across participating schools organized on-site training sessions for classroom teachers to explain the purpose of the survey, precautions, ethical norms, and operational steps. They also sent the link to the online test and the informed consent form to the classroom teachers. In the third step, because the students were studying at home, the classroom teachers informed the parents about the purpose of the survey, precautions, ethical norms, and operational steps through WeChat groups, and sent the link to the online survey and informed consent form to the parents to receive student consent to participate. After parents confirmed both student and parent consent to take the test via the WeChat groups, the students completed the survey independently. Teachers emphasized that students should fill in the questionnaire according to their recent reality. In the fourth step, when school resumed, students brought the signed consent forms to school. The online testing platform specified that all options had to be completed, with assigned value ranges. Students could only submit their answers after confirming that they had completed everything correctly.

## Research tools

### A questionnaire on demographic information

The demographic information surveyed for this study included age, sex, grade, class, family’s marital status, family’s self-assessed economic status, and only-child status. Body mass index (BMI) was calculated from the adolescents’ height and weight. Adolescent obesity was determined according to the screening criteria for overweight and obesity in school-aged children and adolescents (WS/T 586–2018). Studies have shown that respondents’ self-reported height and weight have high predictive power in identifying obesity and can be used in relevant studies [20].

### Questions related to the COVID-19 pandemic

The research team for this project first conducted a literature review on the COVID-19 pandemic, organized the relevant content, established the questionnaire pool, recruited five child and adolescent mental health experts to discuss issues related to adolescent obesity during the COVID-19 pandemic, and developed the initial questionnaire. The team then conducted the pre-survey, which was screened to form the questionnaire.

Question A was a yes/no question asking whether the adolescents had been quarantined at home. Question B asked about the level of fear of COVID-19, with four response options: not afraid, slightly afraid, somewhat afraid, and extremely afraid. Question C asked how often one would use eating behaviors to relieve stress during the pandemic, with four response options: rarely, occasionally, somewhat frequently, and frequently. Question D asked how much time was spent using electronic devices, such as mobile phones or tablets, to keep up with news and information about COVID-19 during the pandemic (referred to as time spent with COVID-19 information). Four response options were available: < 30 min, 30–59 min, 1–3 h, and > 3 h.

### The Simplified Coping Style Questionnaire (SCSQ)

The scale was developed by Xie and Zhang and consists of 20 questions with four response options: never, sometimes, sometimes, and often. The scale is divided into two factors—positive and negative coping styles, respectively—with a test-retest reliability of 0.89, an alpha coefficient of 0.90, a positive coping style coefficient of 0.89, and a negative coping style coefficient of 0.78 [21]. Total scale scores are expressed using coping tendencies. An individual’s coping tendency is calculated by subtracting the negative coping criterion score from the positive coping criterion score; a result greater than zero indicates a positive coping tendency, and a result less than zero indicates a negative coping tendency. This scale has been used in several studies ([22]– [23]).

### An anxiety assessment questionnaire

An anxiety assessment questionnaire was selected from the Mental Health Questionnaire for Secondary School Students compiled by Jisheng Wang. It consists of 10 subscales with 60 items to measure secondary school students’ mental health through self-assessment by test takers on the real situations of their recent mental state. Wang had administered the scale to a sample of 20,000 respondents, and the results showed that the correlation between the 60 items of the scale and the total score ranged from 0.4 to 0.76, showing good values of reliability and validity. Among the items, the factors used to measure anxiety were 6, 15, 34, 43, 46, and 56, which reflected the respondent’s nervousness, sense of insecurity, and unexplained fear. Each item is rated on a 5-point Likert scale, where 1 = none, 2 = mild, 3 = moderate, 4 = quite severe, and 5 = severe [24]. This scale has been used in several studies [25].

### Statistical analysis

SPSS 26.0 was performed using IBM SPSS Statistics for Windows, version 26.0. Descriptive statistical analyses were performed using frequency counts, proportions, and means plus or minus standard deviation, and comparisons of proportions between groups were performed using the *χ2* test, independent samples *t-test*, or analysis of variance. Pearson’s correlation was used to analyze the factors associated with obesity in adolescents. *P* < 0.05 was considered a statistically significant difference. Logistic regression was used to analyze the influencing factors of adolescent obesity. In the logistic regression model, the assignment of independent variables was as follows: sex (1 = boy, 2 = girl), parent’s marital status (1 = normal, 2 = single parents or remarried), only child in the family (1 = yes, 2 = no), self-evaluation of the family’s economic situation (1 = very poor, 2 = poorer, 3 = moderate, 4 = more rich, 5 = very rich), fearful of COVID-19 (1 = not afraid, 2 = slightly afraid, 3 = somewhat afraid, 4 = extremely afraid), the amount of time spent on COVID-19 information (1 = less than 30 min, 2 = 30–59 min, 3 = 1–3 h, 4 = more than 3 h), undergone lockdown or home quarantine (0 = no, 1 = yes), coping tendency (1 = negative coping tendency, 2 = positive coping tendency), using eating behavior to relieve pressure during the COVID-19 pandemic (1 = rarely, 2 = occasionally, 3 = somewhat frequently, 4 = frequently). The measurement data were age, positive and negative coping styles, and. The present study hypothesized that an interactive relationship exists between coping tendency and time spent on COVID-19 information. The amount of time spent on COVID-19 information (reverse; 1 = more than 3 h, 2 = 1–3 h, 3 = 30–59 min, 4 = less than 30 min), coping tendency (1 = negative coping tendency, 2 = positive coping tendency). Obesity was the dependent variable (0 = no, 1 = yes). Variables entered the equation by way of forward.

## Results

### Comparison of factors associated with the COVID-19 pandemic in obese and non-obese adolescents

The survey showed that the obesity rate of boys was significantly higher than that of girls(*P* < 0.05). The age and positive coping style scores of obese adolescents were both significantly lower(*P* < 0.05), and the obesity rate of negative coping tendency was significantly higher than that of positive coping tendency(*P* < 0.05). The group that was extremely afraid of COVID-19 had a significantly higher obesity rate than the other groups(*P* < 0.05). In addition, adolescents who spent more than three hours on COVID-19 information had a significantly higher obesity rate than the other groups(*P* < 0.05). No significant differences were found in obesity rates among adolescents in the presence or absence of anxiety, eating patterns, or home quarantine, nor did obese and non-obese adolescents differ in their scores for negative coping styles (see Table 1).

**Table 1:** Comparison of factors associated with the COVID-19 pandemic in obesity and Non-obesity adolescents (*n* = 13374)

Characteristics	Total *n* = 13,374	Obesity *n* = 2942(22)	Non- Obesity *n* = 10,432(78)	t-test/ chi-square	*P*
Student’s Gender, *N* (%)				75.59	0.000
Boy	6745 (50.4)	1692 (25.1)	5053 (74.9)		
Girl	6629 (49.6)	1250 (18.9)	5379 (81.1)		
Student’s Age (years), *N* (%)	13,374(100)	14.9 ± 1.4	15.3 ± 1.4	11.626	0.000
Positive coping style, M (SD)	13,374(100)	20.8 ± 7.5	21.6 ± 7.2	5.570	0.000
Negative coping style, M (SD)	13,374(100)	10.4 ± 5.3	10.3 ± 5.1	−0.980	0.327
Coping tendency, *N* (%)					
Positive coping tendency	6349(47.5)	1274(20.1)	5057(79.9)	26.287	0.000
Negative coping tendency	7025(52.5)	1668(23.7)	5357(76.3)		
Fearful of COVID-19, *N* (%)				25.160	0.000
Not fearful	3895(29.1)	889 (22.8)	3006 (77.2)		
Slightly fearful	7300(54.6)	1560 (21.4)	5740 (78.6)		
Somewhat fearful	1737(13.0)	357(20.6)	1380 (79.4)		
Extremely fearful	442(3.3)	136(30.8)	306 (69.2)		
The amount of time spent on COVID-19 information, *N* (%)				44.886	0.000
Less than 30 min	5857(94.5)	1158 (19.8)	4699 (80.2)		
30–59 min	5193 (0.6)	1169 (22.5)	4024 (77.5)		
1 to 3 h	1541 (1.1)	404 (26.2)	1137 (73.8)		
More than 3 h	783 (1.9)	211 (26.9)	572(73.1		
Undergone lockdown or home quarantine, *N* (%)				1.278	0.258
No	10,300(77)	2243 (21.8)	8057 (78.2)		
Yes	3074(23)	699 (22.7)	2375 (77.3)		
Anxiety, *N* (%)				1.772	0.183
No	7516	1685 (22.4)	5831 (77.6)		
Yes	5858	1257 (21.5)	4601 (78.5)		
Using eating behavior to relieve pressure during the COVID-19 pandemic, *N* (%)				7.515	0.057
Seldom	3786(28.3)	846 (22.3)	2940 (77.7)		
Occasionally	3840(28.7)	817 (21.3)	3023 (78.7)		
Somewhat frequently	3114(23.3)	357(21.0)	2459 (79.0)		
Frequently	2634(19.7)	624(23.7)	2010 (76.3)		

### Analysis of the correlation between COVID-19 factors and adolescent obesity

This study found that the amount of time spent per day with COVID-19 information was significantly positively correlated with adolescent obesity(*P* < 0.05). Coping tendency and positive coping styles were significantly negatively correlated with adolescent obesity(*P* < 0.05). Fear of COVID-19, quarantine experiences, negative coping styles, anxiety, and eating behavior were not significantly correlated with obesity (see Table 2).

**Table 2:** The correlation between COVID-19 factors and adolescent obesity(*N* = 13374) (*r*,* p*)

Variable	1	2	3	4	5	6	7	8	9
1.Fearful of COVID-19	1	0.125 (0.000)	−0.025 (0.004)	0.000 (0.995)	−0.005 (0.545)	0.004(0.672)	0.073 (0.000)	0.005 (0.548)	0.005 (0.567)
2.The amount of time spent on COVID-19 information		1	0.002 (0.829)	0.066(0.000)	0.021 (0.015)	0.031(0.000)	−0.012 (0.167)	0.017 (0.048)	0.057 (0.000)
3.Undergone lockdown or home quarantine			1	0.010 (0.260)	0.038 (0.000)	−0.019(0.28)	0.03 (0.000)	0.027 (0.002)	0.010 (0.258)
4.Positive coping style				1	0.321 (0.000)	0.442(0.000)	−0.25 (0.000)	0.234 (0.000)	−0.049 (0.000)
5.Negative coping style					1	−0.486(0.000)	0.190 (0.000)	0.606 (0.000)	0.009 (0.319)
6.Coping tendency						1	−0.336 (0.000)	−0.268(0.000)	−0.044(0.000)
7.Anxiety							1	0.082(0.000)	−0.002 (0.183)
8.Using eating behavior to relieve pressure during the COVID-19 pandemic								1	0.008 (0.378)
9. Obesity									1

### Multielement analysis of the obesity of adolescents from a variety of factors

According to the regression analysis of adolescents categorized as obese, the protective factors that were identified were female sex (0.678, 0.624–0.738), age (0.847,0.823–0.872), self-evaluation of the family’s economic situation (0.905, 0.840–0.976), and positive coping style (0.980, 0.974–0.986) (*P* < 0.05). The risk factors that were identified were the amount of time spent on COVID-19 information (1.140, 1.088–1.195) and the use of eating behaviors to relieve pressure during the COVID-19 pandemic (1.084, 1.042–1.127) (*P* < 0.05) (see Table 3).

**Table 3:** Multi-element analysis of the obesity of adolescents from a variety of factors(*N* = 13374)

Characteristic	AOR(95% C.I.)	*P*
age	0.847(0.823–0.872)	0.000
girl	0.678(0.624–0.738)	0.000
self-evaluation of the family’s economic situation	0.905(0.840–0.976)	0.009
the amount of time spent on COVID-19 information	1.140(1.088–1.195)	0.000
using eating behavior to relieve pressure during the COVID-19 pandemic	1.084(1.042–1.127)	0.000
positive coping style	0.980(0.974–0.986)	0.000

sex,1 = boy,2 = gir; self-evaluation of the family’s economic situation:1 = very poor,2 = poorer,3 = moderate,4 = more rich,5 = very rich; the amount of time spent on COVID-19 information:1 = less than 30 min, 2 = 30–59 min, 3 = 1–3 h, 4 = more than 3 h; using eating behavior to relieve pressure during the COVID-19 pandemic,1 = rarely,2 = occasionally,3 = somewhat frequently, 4 = frequently.The age, positive coping style is measurement data.Dependent variable is obesity,0 = no,1 = yes

### Interaction of adolescent positive coping tendency and the amount of time spent on COVID-19 information(reverse) on obesity

To further investigate the role of positive coping tendency and less time focus on COVID-19 information in obesity, taking into account the collinearity between variables, this study selected age, only-child status, family’s marital status, and self-ev`aluation of the family’s economic situation as adjusting factors and incorporated them into the logistic regression model, along with coping tendency, the amount time focus on COVID-19 information, and the interaction between the multiplication of positive coping tendency and the less time focus on COVID-19 information. These findings indicate that the interaction between positive coping tendency and less time focus on COVID-19 information exerts a significant influence on adolescent obesity (0.923, 0.904–0.942) (*P* < 0.05) (see Table 4).

**Table 4:** Interaction of adolescent positive coping tendency and the amount of time spent on COVID-19 information(reverse) on obesity(*N* = 13374)

Characteristic	AOR(95% C.I.)	*P*
age	0.845(0.821–0.870)	0.000
girl	0.699(0.643–0.760)	0.000
self-evaluation of the family’s economic situation	0.893(0.829–0.962)	0.003
positive coping tendency*the amount of time spent on COVID-19 information**(reverse)**	0.923(0.904–0.942)	0.000

sex,1 = boy,2 = girl; self-evaluation of the family’s economic situation:1 = very poor,2 = poorer,3 = moderate,4 = more rich,5 = very rich; The age, positive coping style, negative coping style and anxiety is measurement data. The amount of time spent on COVID-19 information(reverse):1 = more than 3 h, 2 = 1–3 h,3 = 30–59 min, 4 = less than 30 min; coping tendency,1 = negative coping tendency,2 = positive coping tendency.Dependent variable is obesity,0 = no,1 = yes

## Discussion

This study was a large-sample survey conducted during the COVID-19 pandemic to comprehensively investigate the time spent on COVID-19 information, coping styles, anxiety, and eating patterns among adolescents, and to further elucidate the mechanisms underlying adolescent obesity during crises. This study showed that the obesity rate among adolescents during the COVID-19 pandemic lockdowns was 22%, higher than the rate reported in China in 2020 for adolescents aged 6–17 years, which fell to 19% [26]. The present study identified several protective factors in adolescents with obesity. These factors were female sex, age, self-evaluation of the family’s economic situation, and positive coping style. The risk factors that were identified included the amount of time spent on COVID-19 information and the use of eating behaviors to relieve pressure during the COVID-19 pandemic. Concurrently, the findings indicated that the interaction between positive coping tendency and less time focus on COVID-19 information exerts a significant influence on adolescent obesity.

Specifically, many studies have found a significant increase in obesity rates among adults [6, 8, 27], and scholars from Korea [27], Greece [28], and Germany [29] have reported increases in children’s weight during the COVID-19 pandemic. A survey of 10,082 youth in China found an increase in obesity from 10.5% to 12.6% during the COVID-19 lockdown [9]; another study in China that included 19,066 preschoolers found an increase in childhood obesity from 10.47% to 12.28% before and after school closure [8]. Significant weight gain during the pandemic was also found in Chinese adults [30]. This study is consistent with previous studies in other age groups.

In this study, we found that female sex, age, and self-assessed economic status are protective factors against adolescent obesity and can reduce its incidence. We have discussed these conclusions in the relevant literature [31]. First, during the COVID-19 pandemic, adolescents reduced activities outside the home to maintain social distance and mainly studied online at home; this reduction in activities thus led to obesity [7, 32]. However, the findings of this study suggest that a brief period of home isolation does not significantly affect the prevalence of adolescent obesity. One potential explanation for this phenomenon is that the duration of the home quarantine period is relatively brief, which has minimal impact on the eating and activity habits of adolescents. Furthermore, the survey was conducted two years after the onset of the epidemic, at which point it was found that adolescents had become less sensitive to epidemic-related measures, thereby resulting in a relatively minor impact on their obesity levels. Casanova found no causal association between BMI and anxiety disorders in a one- and two-sample Mendelian randomization analysis of 145,668 European participants in the United Kingdom (*p* = 0.60) [33]. The present study demonstrates that trepidation regarding epidemics, anxiety, and emotional eating behavior is not associated with an increased prevalence of obesity in adolescents.

In the present study, a positive correlation was identified between obesity and the time spent focusing on the pandemic, this suggests that adolescents who expend excessive time consuming crisis information during a crisis may be vulnerable to adverse consequences, particularly with respect to obesity. The study showed that positive coping styles had a significant protective effect on adolescent obesity, and the higher the adolescent’s positive coping style score, the lower the odds of obesity. Research indicates a strong correlation between the adoption of a positive coping style and the maintenance of a healthy lifestyle during the COVID-19 pandemic [34]. A positive way for adolescents to cope may be to reduce obesity by promoting healthy lifestyles. The present study did not find a role of negative coping styles in adolescent obesity, which is inconsistent with previous findings [16, 17]. This study further suggests that focusing on positive coping styles may contribute to reducing the incidence of adolescent obesity when developing intervention programs to address this issue.

The biosocioecological model posits that adolescent attitudes towards obesity and screen-related behaviors significantly impact obesity levels. The present study demonstrates that a positive coping tendency coupled with less time focus on COVID-19 information can act as a mutual reinforcer in the process of reducing adolescent obesity; consequently, adolescents who exhibit positive coping tendency are more likely to reduce the incidence of adolescent obesity when they allocate less time to COVID-19 information about the epidemic, which has not been demonstrated in previous research. One potential explanation for this phenomenon is that adolescents who exhibit both positive coping tendencies and a reduced focus on COVID-19 information may adopt healthier lifestyles, thereby leading to a reduction in the prevalence of obesity. This study indicates the need for a systematic examination of the synergistic effects of adolescents’ coping tendency and the focus on time-to-crisis information when developing obesity intervention programs for adolescents in future chronic crises.

## Limitations

Although this study found an association between adolescents’ time spent on COVID-19 information and obesity, it cannot prove a causal relationship because it is a cross-sectional study; relevant longitudinal results are needed for further validation. Additionally, the data were collected between April and June 2022. Notwithstanding the fact that students have been engaged in remote learning for two years, the COVID-19 pandemic continues unabated. It is evident that adolescents’ sensitivity to information about the epidemic has diminished. Furthermore, the coping strategies exhibited by adolescents during the initial phase of popularity differ from those observed in the contemporary period. It is important to note that these factors may affect the conclusions of this study. Moreover, the self-report method used in this study to conduct the survey did not strictly investigate adolescents’ weight or the time that they spent reading COVID-19 information on mobile phones, which results in some bias. Additionally, this study used convenience sampling to examine a population of Chinese adolescents, which affected the generalizability of the findings. Future studies should use more objective or interventional methods to demonstrate the relationship between information attention and obesity.

## Conclusions

This study found that increased daily time spent with COVID-19 information was associated with a higher incidence of adolescent obesity. Positive coping styles have been observed to reduce the incidence of obesity in adolescents. Positive coping tendency and less time focus on COVID-19 information can mutually reinforce the reduction in adolescent obesity. This study further revealed the mechanisms underlying the factors that affect adolescent obesity, providing a reference for improving the theoretical framework of adolescent obesity. In developing interventions for adolescent obesity during periods of chronic crisis, authorities must focus on enhancing adolescents’ coping tendency and reducing the amount of time spent focusing on crises.

## Supplementary Information

Supplementary Material 1.

Supplementary Material 2.

Supplementary Material 3.

Supplementary Material 4.

Supplementary Material 5.

## Data Availability

Due to privacy of participants, please ask for corresponding author for research data.

## Abbreviations

BMI: Body mass index
CI: Confidence interval
SE: Standard Error
AOR: Adjusted Odds Ratio
