# Supplementary material for: The interactive roles between coping tendency and focus on COVID-19 information time in Adolescent Obesity
Source: BMC Psychol. 2025 Dec 11;14:68. doi: 10.1186/s40359-025-03766-x (PMC12801824; doi:10.1186/s40359-025-03766-x)
Supplement: Supplementary file 1 — Supplementary Material 1. [file 40359_2025_3766_MOESM1_ESM.pdf]

**Questionnaire survey on adolescent physical and mental health**

Dear Fellow Students: Hello! Physical and mental health is the foundation for students to successfully complete their studies and achieve their dreams. To better promote the development of students physical and mental health, please fill out the following survey questionnaire. The answers are not about right or wrong, just be honest and fill them out truthfully. The overall results will be used to guide the implementation of mental health education for students. We will strictly keep all related information confidential. You can exit at any time during the process. Thank you for your cooperation!

Do you agree to participate in this questionnaire?

○ agree
○ Not agree (please go to the end of the questionnaire and submit your answer)

1. Number: (Please fill in the corresponding number that has been informed to you. If not informed to you, please fill in 101)

____________________

2.Your grade

| | | | | |
|---|---|---|---|---|
| ○ Grade 1 | ○ Grade 2 | ○ Grade 3 | ○ Grade 4 | ○ Grade 5 |
| ○ Grade 6 | ○ Grade 7 | ○ Grade 8 | ○ Grade 9 | ○ Grade 10 |
| ○ Grade 11 | ○ Grade 12 | | | |

3.Your class

| | | | | |
|---|---|---|---|---|
| ○1 | ○2 | ○3 | ○4 | ○5 |
| ○6 | ○7 | ○8 | ○9 | ○10 |
| ○11 | ○12 | ○13 | ○14 | ○15 |
| ○16 | ○17 | ○18 | ○19 | ○20 4, |

4.your age (years old):________________________________________

5.Your gender:

○ A, male
○ B, female

6. How long you have lived in Tianjin:

○ A. Migrating to Tianjin from other places for less than one year, but not including one year
○ B. Those who have moved to Tianjin from other places and have lived there for one to three years, excluding the three years
○ C. You have moved to Tianjin from other places and have lived

there for at least three years or you are a local resident of Tianjin

7. Are you an only child?

○ A, yes
○ B, deny

8. You feel the economic pressure of your family:

○ A. Very difficult. The pressure is great

○ B, more difficult, more pressure
○ C. Generally no pressure
○ D. More affluent and superior
○ E. Very rich and superior

9. Your parents marital status:

○ A. Normal family
○ B. Remarried families
○ C, parent family

10. Your boarding and day attendance situation in the last half semester:

○ A, in residence
○ B, attend a day school

11.Height (m): _____

12, weight (KG):_____

13. How afraid are you of the current COVID-19 pandemic?

○ not afraid

○ slightly afraid

○ somewhat afraid

○ extremely afraid

14. How much time do you spend on the media such as mobile or TV every day in the past three months to follow the COVID-19 pandemic?

○ Less than 0.5 hours
○ 0.5-1 hour (excluding 1 hour)
○ 1-3 hours (excluding 3 hours)
○ More than 3 hours

15. Have you been quarantined in a hotel, home or community for the past three months due to the COVID-19 pandemic?

○ yes
○ no

16. In the past three months during the COVID-19 pandemic, how often have you used eating to relieve stress?

○ Rarely

○ Occasionally

○ Somewhat frequently

○ Frequently
