# Supplementary material for: The interactive roles between coping tendency and focus on COVID-19 information time in Adolescent Obesity
Source: BMC Psychol. 2025 Dec 11;14:68. doi: 10.1186/s40359-025-03766-x (PMC12801824; doi:10.1186/s40359-025-03766-x)
Supplement: Supplementary file 2 — Supplementary Material 2. [file 40359_2025_3766_MOESM2_ESM.docx]

Table 1 Comparison of Factors Associated with the COVID-19 pandemic in Obesity and Non-obesity Adolescents (n=13374)

| Characteristics | Total  n=13374 | Obesity  n=2942(22) | Non- Obesity  n=10432(78) | t-test/  chi-square | P |
| --- | --- | --- | --- | --- | --- |
| Student’s Gender, N (%) |  |  |  | 75.592^***^ | 0.000 |
| Boy | 6745 (50.4) | 1692 (25.1) | 5053 (74.9) |  |  |
| Girl | 6629 (49.6) | 1250 (18.9) | 5379 (81.1) |  |  |
| Student’s Age (years), N (%) | 13374（100） | 14.94±1.442 | 15.28±1.421 | 11.626^***^ | 0.000 |
| Positive coping style, M (SD) | 13374（100） | 20.760±7.448 | 21.619±7.167 | 5.570^***^ | 0.000 |
| Negative coping style, M (SD) | 13374（100） | 10.389±5.293 | 10.282±5.134 | -0.980 | 0.327 |
| Coping tendency, N (%) |  |  |  |  |  |
| Positive coping tendency | 6349（47.5） | 1274（20.1） | 5057（79.9） | 26.287 | 0.000 |
| Negative coping tendency | 7025（52.5） | 1668（23.7） | 5357（76.3） |  |  |
| Fearful of COVID-19, N (%) |  |  |  | 25.160^***^ | 0.000 |
| Not fearful | 3895（29.1） | 889 (22.8) | 3006 (77.2) |  |  |
| Slightly fearful | 7300（54.6） | 1560 (21.4) | 5740 (78.6) |  |  |
| Somewhat fearful | 1737（13.0） | 357(20.6) | 1380 (79.4) |  |  |
| Extremely fearful | 442（3.3） | 136(30.8) | 306 (69.2) |  |  |
| The amount of time spent on COVID-19 information, N (%) |  |  |  | 44.886^***^ | 0.000 |
| Less than 30 minutes | 5857(94.5) | 1158 (19.8) | 4699 (80.2) |  |  |
| 30-59 minutes | 5193 (0.6) | 1169 (22.5) | 4024 (77.5) |  |  |
| 1 to 3 hours | 1541 (1.1) | 404 (26.2) | 1137 (73.8) |  |  |
| More than 3 hours | 783 (1.9) | 211 (26.9) | 572(73.1 |  |  |
| Undergone lockdown or home quarantine, N (%) |  |  |  | 1.278 | 0.258 |
| No | 10300（77） | 2243 (21.8) | 8057 (78.2) |  |  |
| Yes | 3074（23） | 699 (22.7) | 2375 (77.3) |  |  |
| Anxiety, N (%) |  |  |  | 1.772 | 0.183 |
| No | 7516 | 1685 (22.4) | 5831 (77.6) |  |  |
| Yes | 5858 | 1257 (21.5) | 4601 (78.5) |  |  |
| Using eating behavior to relieve pressure during the COVID-19 pandemic, N (%) |  |  |  | 7.515 | 0.057 |
| Seldom | 3786（28.3） | 846 (22.3) | 2940 (77.7) |  |  |
| Occasionally | 3840（28.7） | 817 (21.3) | 3023 (78.7) |  |  |
| Somewhat frequently | 3114（23.3） | 357(21.0) | 2459 (79.0) |  |  |
| Frequently | 2634（19.7） | 624(23.7) | 2010 (76.3) |  |  |

*^*^ p < 0.05; ^**^ p < 0.01; ^***^ p < 0.001.*
