# Supplementary material for: The interactive roles between coping tendency and focus on COVID-19 information time in Adolescent Obesity
Source: BMC Psychol. 2025 Dec 11;14:68. doi: 10.1186/s40359-025-03766-x (PMC12801824; doi:10.1186/s40359-025-03766-x)
Supplement: Supplementary file 3 — Supplementary Material 3. [file 40359_2025_3766_MOESM3_ESM.docx]

## Table 2 The correlation between COVID-19 factors and adolescent obesity(N = 13374) (*r,p*)

| Variable | 1 | 2 | 3 | 4 | 5 | 6 | 7 | 8 | 9 |
| --- | --- | --- | --- | --- | --- | --- | --- | --- | --- |
| 1.Fearful of COVID-19 | 1 | 0.125^***^(0.000) | -0.025^*^  (0.004) | 0.000  (0.995) | -0.005  (0.545) | 0.004（0.672） | 0.073^***^  (0.000) | 0.005  (0.548) | 0.005  (0.567) |
| 2.The amount of time spent on COVID-19 information |  | 1 | 0.002  (0.829) | 0.066^***^(0.000) | 0.021^*^  (0.015) | 0.031^***^（0.000） | -0.012  (0.167) | 0.017^*^  (0.048) | 0.057^***^  (0.000) |
| 3.Undergone lockdown or home quarantine |  |  | 1 | 0.010  (0.260) | 0.038^***^(0.000) | -0.019（0.28） | 0.030^***^  (0.000) | 0.027^**^  (0.002) | 0.010  (0.258) |
| 4.Positive coping style |  |  |  | 1 | 0.321^***^(0.000) | 0.442^***^（0.000） | -0.252^***^  (0.000) | 0.234^***^(0.000) | -0.049^***^  (0.000) |
| 5.Negative coping style |  |  |  |  | 1 | -0.486^***^（0.000） | 0.190^***^  (0.000) | 0.606^***^(0.000) | 0.009  (0.319) |
| 6.Coping tendency |  |  |  |  |  | 1 | -0.336^***^（0.000） | -0.268^***^（0.000） | -0.044^***^（0.000） |
| 7.Anxiety |  |  |  |  |  |  | 1 | 0.082^***^(0.000) | -0.002  (0.183) |
| 8.Using eating behavior to relieve pressure during the COVID-19 pandemic |  |  |  |  |  |  |  | 1 | 0.008  (0.378) |
| 9. Obesity |  |  |  |  |  |  |  |  | 1 |

*^*^ p < 0.05; ^**^ p < 0.01; ^***^ p < 0.001.*
