# Supplementary material for: The interactive roles between coping tendency and focus on COVID-19 information time in Adolescent Obesity
Source: BMC Psychol. 2025 Dec 11;14:68. doi: 10.1186/s40359-025-03766-x (PMC12801824; doi:10.1186/s40359-025-03766-x)
Supplement: Supplementary file 4 — Supplementary Material 4. [file 40359_2025_3766_MOESM4_ESM.docx]

Table 3 Multi-element analysis of the obesity of adolescents from a variety of factors(N = 13374)

| Characteristic | *AOR(95% C.I.)* | *P value* |
| --- | --- | --- |
| age | 0.847(0.823-0.872) | 0.000 |
| girl | 0.678(0.624-0.738) | 0.000 |
| self-evaluation of the family's economic situation | 0.905(0.840-0.976) | 0.009 |
| the amount of time spent on COVID-19 information | 1.140(1.088-1.195) | 0.000 |
| using eating behavior to relieve pressure during the COVID-19 pandemic | 1.084(1.042-1.127) | 0.000 |
| positive coping style | 0.980(0.974-0.986) | 0.000 |

*^*^ p < 0.05; ^**^ p < 0.01; ^***^ p < 0.001.*
