# Supplementary material for: The interactive roles between coping tendency and focus on COVID-19 information time in Adolescent Obesity
Source: BMC Psychol. 2025 Dec 11;14:68. doi: 10.1186/s40359-025-03766-x (PMC12801824; doi:10.1186/s40359-025-03766-x)
Supplement: Supplementary file 5 — Supplementary Material 5. [file 40359_2025_3766_MOESM5_ESM.docx]

Table 4 Interaction of adolescent positive coping tendency and the amount of time spent on COVID-19 information(reverse) on obesity(N = 13374)

| Characteristic | *AOR(95% C.I.)* | *P value* |
| --- | --- | --- |
| age | 0.845(0.821-0.870) | 0.000 |
| girl | 0.699(0.643-0.760) | 0.000 |
| self-evaluation of the family's economic situation | 0.893(0.829-0.962) | 0.003 |
| positive coping tendency*the amount of time spent on COVID-19 information**(reverse)** | 0.923(0.904-0.942) | 0.000 |
